# Habitat selection patterns of a species at the edge – case study of the native racer goby population in Central Europe

**DOI:** 10.1038/s41598-019-56264-7

**Published:** 2019-12-23

**Authors:** Krzysztof Kukuła, Bernadetta Ortyl, Aneta Bylak

**Affiliations:** 1 Department of Ecology and Environmental Protection, University of Rzeszow, Zelwerowicza 4, 35-601 Rzeszow, Poland; 2 Department of Nature Conservation and Landscape Ecology, University of Rzeszow, Zelwerowicza 4, 35-601 Rzeszow, Poland

**Keywords:** Freshwater ecology, Invasive species

## Abstract

Invasive alien species are regarded a nuisance. This extends into a lack of conservation efforts in their native range. As a consequence, conservation of e.g. range-edge populations is neglected. Gobiidae have many representatives of alien species in European freshwaters, and therefore they have a bad reputation. Objectives of this study were to: define the habitat selection patterns of a species at the edge, and examine the ontogenetic variation in its distributions, i.e. spatial distribution of different size classes. A racer goby *Babka gymnotrachelus* (syn. *Neogobius gymnotrachelus*) population was selected for the model. In numerous European river basins, Ponto-Caspian racer goby has been an invasive alien species of interest to researchers for many years. Recently, however, native populations of the species have been described in the Polish tributary of the upper Dniester River (Black Sea basin). We used habitat data and densities of racer goby to disentangle the habitat selection patterns of the species at a river reach at the edge of its native range. Evident preferences towards habitats with large submerged objects serving as hiding places were characteristic of the largest gobies. Adult, largest gobies were very likely to choose the ‘boulders’ site, while forcing smaller individuals to occupy places with faster water current, i.e. less suitable in terms of saving energy. At a larger geographic scale, a significant portion of the submountain river was unsuitable for racer gobies. At the edge of the racer goby range, patches providing habitats suitable for the species were scarce and scattered. With regard to invasive populations, the presence of stony bottoms, quite certainly cannot be considered as a factor excluding potential colonisation by racer goby, and in submountain rivers it might be the preferred kind of bottom.

## Introduction

Non-native and translocated freshwater fish species are one of the most serious problems of riverine ichthyofauna conservation^1–3^. There are examples of invasive species altering the native species by competitive exclusion, niche displacement, hybridization, introgression, predation, and ultimately extinction^4–6^. Sometimes, alien species of economic or fishing importance are more protected than native species, for example brown trout (*Salmo trutta*) in New Zealand^7^. On the other hand, recognising a species as alien invasive in many areas almost immediately results in labelling it as ‘invasive’ and as a redundant component of fish fauna anywhere it is recorded^8^. It is worth emphasizing that we might be missing planning conservation of such species in their native range of occurrence. Some families of fishes, such as gobies (Gobiidae), have many representatives of alien species in European freshwaters, and therefore have a bad reputation. Round goby (*Neogobius melanostomus* (Pallas, 1814)), monkey goby (*Neogobius fluviatilis* (Pallas, 1811)), tubenose goby (*Proterorhinus semilunaris* (Pallas, 1814)), and racer goby (*Babka gymnotrachelus* (Kessler, 1857), a species separated from the genus *Neogobius* and reclassified into the new genus *Babka*^9^) invaded the European river systems from Pripyat, Bug, across the Vistula, and the Danube Rivers^8^. In Germany, Slovakia, the Czech Republic, Romania, Serbia, or in Poland in the Vistula River basin, gobies are considered invasive^10–16^.

As an invasive alien species, racer goby has been of interest to researchers for many years (e.g.^8,13,17,18^). Nonetheless, the expansion and establishment patterns of racer goby have been investigated to the lowest degree among Gobiidae^19,20^, and data regarding its ability to colonise lotic environments are still scarce^21^. Since the mid 1990s, racer goby has invaded several central European rivers. Invasive racer gobies were recorded in Poland in 1995 in the Bug River^22^, and in 2000 in the lower Vistula River^23^, and its fast spreading in Vistula was observed in 2002^24^. An invasion of racer goby has also been recently described in Lithuania, probably reached by the species through artificial canals connecting the Neumnas River with the Pripyat and Vistula Rivers^25^ and in Greece, where it was found in the Evros River catchment^26^.

The racer goby is a native and widespread species from the Black, Azov, Marmara, and Caspian Sea basins^27,28^.The Racer goby is native also in the Strwiąż River^29–31^, and falls under the Least Concern category according to the last International Union for Conservation of Nature and Natural Resources (IUCN) Red List assessment^27^. In many publications from the 20th century, racer goby was recognised as an integral element of the freshwater ichthyofauna of the Dniester River basin. The earliest reports of racer goby from Dniester come from the 19th century. The species was described by Kessler (1857) from the Zbruch River which is a Dniester tributary, and the Dniester River basin was recognised as the *locus typicus* for racer goby^29,32^. Historical information on the distribution of racer goby in the upper Dniester River is rather obscure, and includes no exact location data. According to literature, the species was generally common and abundant in the upper Dniester River in the 1940s (e.g.,^30,33^). Some newer papers refer to this fact (e.g.,^19,29^), although this large river flowing into the Black Sea was overlooked in many studies, that focused on the Danube and Dnieper River basins. As a result,information concerning the native and invasive area was sketchy and/or contradictory^20^, and Dniester was sometimes incorrectly marked in maps as a tributary of the Danube River(e.g.,^20^).

**Figure 1:**
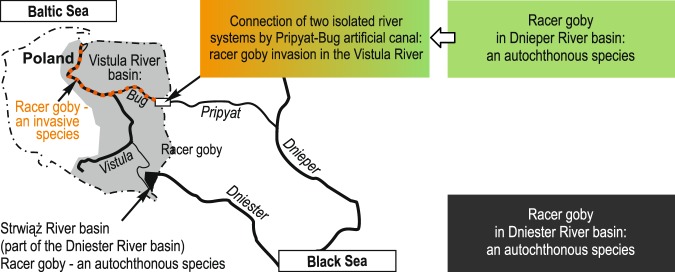
Division of Vistula and Strwiąż River basins, and a simplified model showing the origin of two racer goby (*Babka gymnotrachelus*) populations in Polish inland waters.

With regard to racer goby, the postulate of its double origin and therefore its dual nature (alien invasive, and native) in territorial waters in Poland (Fig. 1) has been recently raised. Some populations of racer goby in Poland are invasive (in the Vistula River in the Baltic Sea basin)^12,17,32^, whereas others (inhabiting the tributary of the Dniester River in the Black Sea basin) are native^29,34^. In the Carpathian tributary of the upper Dniester River, namely the Strwiąż River, native populations of this species have been described in 2013^34^. Genetic research of the fish revealed the existence of two genetically different racer goby populations in Polish inland waters: the invasive population inhabiting the Vistula and Western Bug Rivers (source population from the Dnieper River), and a native population found in the Strwiąż River (a tributary of the upper Dniester River)^29^. This native population operates on the edge of its range, and survived in one of the few well-preserved tributaries of the upper Dniester River^35^. Therefore, we think the population in the Strwiąż River is unique. Racer gobies were very abundant in some areas of this submountain river, but they are found in specific habitat patches^35^.

In flowing waters, racer gobies are associated mainly with large rivers and found in well-vegetated or highly complex habitats. The racer goby is considered an opportunistic species^24^ with a broad tolerance for factors such as water temperature and salinity^27^. In its native range, the racer goby is a typical inhabitant of estuaries as well as coastal zones of dam reservoirs^35^. Mountain and submountain racer goby populations have not been described so far, and the species has been often characterised as a lowland fish^36,37^. In rivers, it shows an evident preference for zones with slower water velocities and fine-bottom substrate^38^. In difficult environmental conditions at the edge of the species range, however, other habitat selection patterns are expected. Due to their high mobility, fish at various stages of their life cycle select habitats most suitable for spawning, fry growth, foraging, and/or overwintering^39,40^. Therefore, attention is often paid to the ontogenetic variation in the fish distribution i.e. the spatial distribution of different size classes, and shifts in the selection of one type of habitat to another^41,42^. Due to this, racer gobies may also have other habitat preferences depending on the size class.

The detailed objectives of this study were to (i) define the habitat selection patterns of the racer goby at the edge, and (ii) examine ontogenetic variation in its distributions. Our research was also expected to facilitate forecasting of alien racer goby expansion rates.

## Results

**Figure 2:**
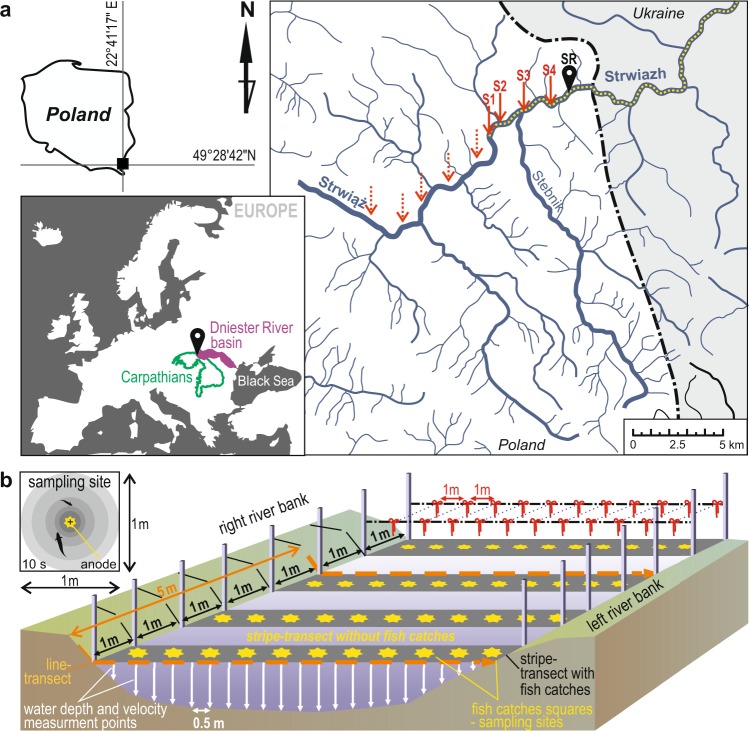
(**a**) Map of the study area showing the river reach surveyed (SR, black pin); dotted line – range of the racer goby (*Babka gymnotrachelus*) in the Strwiąż River basin; dash-dotted line – state border; S1–S4 – additional fish sampling locations; dotted arrows indicate locations where no racer goby was found; (**b**) Schematic depicting field sampling procedures.

The bottom of the river reach consists of boulders and smaller stones, with the remainder consisting of gravel and sand. These substrate fractions show patchy distribution on the river bottom. Based on the bottom substrate fractions (see Supplementary Fig. S1), sampling sites (1 m x1 m squares, Fig. 2) were divided into three main habitat types. In the first habitat type (habitat types were distinguished on the basis of cluster analysis - please see the method section), the dominant bottom substrate fraction was gravel (habitat type: gravel, GR). In the second habitat, the most numerous fractions were stones of various sizes (habitat type: cobbles and pebbles, PE), and the third habitat type was dominated by boulders and large cobbles (habitat type: boulders, BO). Significant differences were observed in the shares of distinguished bottom substrate fractions in these three habitat types. All of the compared pairs of habitat types were significantly different (see Supplementary Table S1), with the largest proportion having gravel, followed by large cobbles and boulders (see Supplementary Table S2).

**Figure 3:**
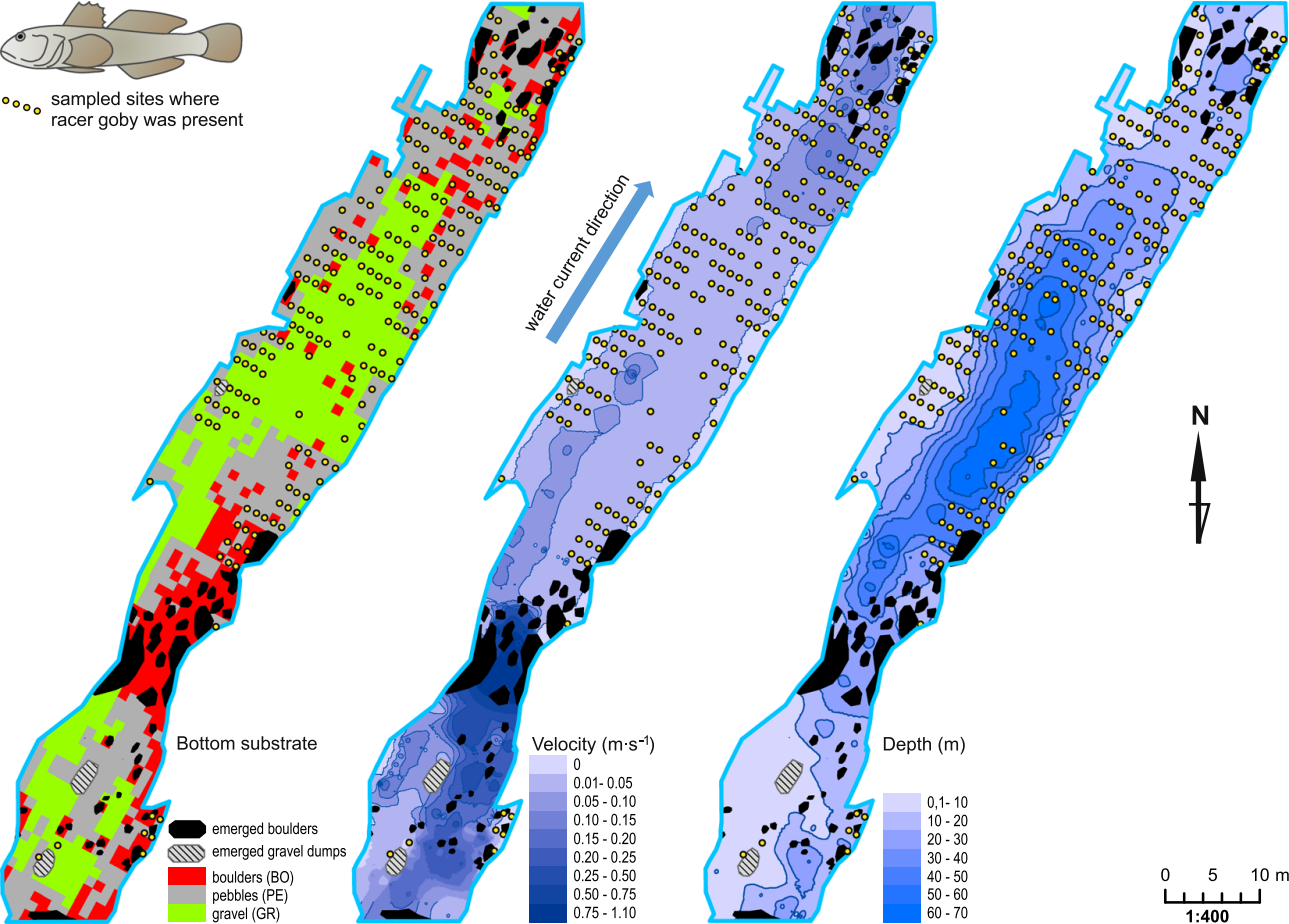
Maps of the bottom substrates, velocities, and water depths of the submountain river reach surveyed (SR).

In the surveyed river reach, shallow fragments were present in zones near the banks and in the upper zone. However, in the middle part of the river reach, deeper regions (depth exceeding 50 cm) were observed. The water current varied; zones with slow flowing or even stagnant water as well as sections with water currents exceeding 1 m s^−1^ were observed. In sections with fast flowing water, boulders dominated the bottom substrate (habitat type: BO) (Fig. 3). The water temperature of the surveyed reach ranged from 18.6 °C to 20.4 °C (SD = 0.66 °C), and the dissolved oxygen content exceeded 9.5 mg L^−1^ (range 9.66–10.03 mg L^−1^, SD = 0.14 mg L^−1^). Water conductivity ranged from 305 μS to 473 μS (SD = 43.9 μS). No statistically significant differences were determined between mean values of water temperature, dissolved oxygen, and water conductivity in three types of habitats.

At four additional sites (S1-S4) above the analysed river reach, racer goby was scarce, and its density varied from 3 to 12 individuals per 100 m2 of the area of the river (Table 1). Site S1 was the highest located fragment of the Strwiąż River where the species was recorded^35^. In the analysed river reach (SR), the density of racer goby was 98 ind. 100 m^−2^. All three size classes of the fish were also abundantly represented (Table 1). A total of 431 racer goby specimens were caught, including 145 belonging to the SF size class, 215 belonging to the MF size class, and 71 large individuals (LF class). The distinguished habitat types differed significantly among the racer goby population size classes (see Supplementary Table S3, Table 2), with the smallest individuals (SF) contributing most to the dissimilarity (see Supplementary Table S4). A considerable habitat overlap exists between SF and MF fishes, but not between LF and the other two size classes (the degrees of habitat overlap (CZ) were as follows: size classes SF *vs*. MF = 0.72; SF *vs*. LF = 0.52; and MF *vs*. LF = 0.58). Notably, only 38 racer gobies (out of all 431 caught) were found in zones where the water current at the bottom exceeded 0.1 m s^−1^.

**Table 1:** Racer goby densities (ind. 100 m^−2^) at locations in the Strwiąż River, in 2015 catches; SF – small fish, total length (Tl) ≤ 40 mm; MF – medium-sized fish, 40 mm < Tl ≤ 60 mm; LF – large fish, Tl > 60 mm; fish sampling locations numbering is consistent with Fig. 2.

Fish sampling locations	Racer goby size categories			All size classes
	SF	MF	LF	
S1	1,1	7,5	0,6	9,2
S2	0,7	1,1	1,5	3,3
S3	0,5	1,0	3,5	5,0
S4	3,1	3,6	5,8	12,5
Surveyed river reach (SR)	32,2	47,8	17,8	97,8

**Table 2:** Summary of generalized linear models (GLM) showing the effects of habitat type (n = 3 categories), current velocity and water depth on racer goby abundance; BO – boulder habitat, PE – pebble habitat (detailed habitat characteristics - Fig. 2, Table A2); fish size categories: SF – small fish, total length (Tl) ≤ 40 mm; MF – medium-sized fish, 40 mm < Tl ≤ 60 mm; LF – large fish, Tl > 60 mm.

Effects	d.f.	Estimate	SE	−95% c.l.	+95% c.l.	Wald test	*P*
**SF**							
Intercept	1	−1.287	0.181	−1.641	−0.933	50.772	0.0000
Current velocity	1	−7.142	1.571	−10.221	−3.819	20.667	0.0000
Depth	1	0.007	0.006	−0.006	0.019	1.098	0.2946
Bottom type	2						
BO		0.157	0.143	−0.122	0.433	1.219	0.2695
PE		0.647	0.127	0.397	0.897	25.716	0.0000
**MF**							
Intercept	1	−0.896	0.147	−1.184	−0.607	37.028	0.0000
Current velocity	1	−6.796	1.319	−9.381	−4.210	26.538	0.0000
Depth	1	0.004	0.005	−0.006	0.014	0.689	0.4064
Bottom type	2						
BO		−0.062	0.128	−0.313	0.188	0.236	0.6272
PE		0. 517	0.106	0.309	0.725	23.735	0.0000
**LF**							
Intercept	1	−1.964	0.243	−2.440	−1.489	65.519	0.0000
Current velocity	1	−9.736	2.773	−15.170	−4.301	12.328	0.0004
Depth	1	0.015	0.007	0.0001	0.029	3.903	0.0482
Bottom type	2						
BO		0.446	0.170	0.114	0.779	6.911	0.0086
PE		0.041	0.184	−2.440	−1.489	0.050	0.8223

**Figure 4:**
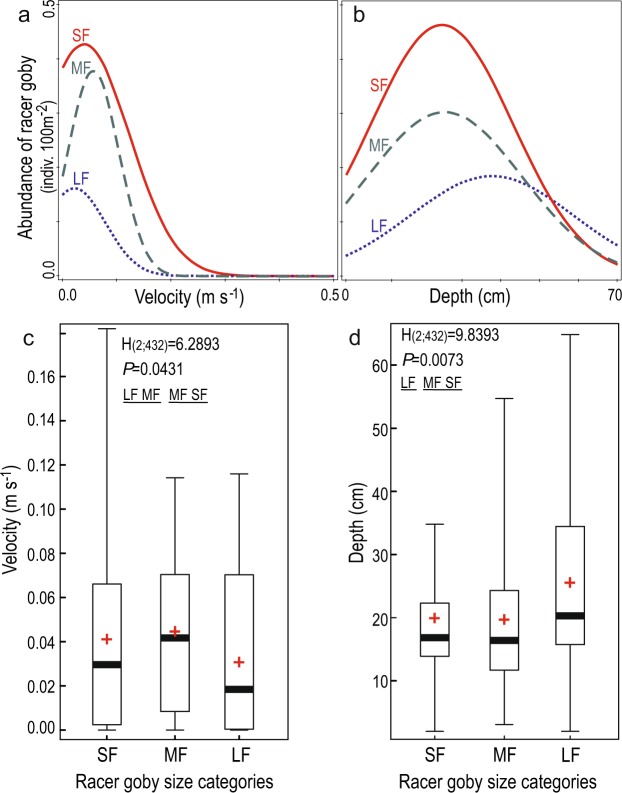
Responses of racer gobies of differing class size abundances - (generalized linear models) to (**a**) velocity and (**b**) depth and nonparametric one-way ANOVA (Kruskal-Wallis test) for comparisons of water current velocities (**c**) and water depth (**d**) at sites where racer gobies were found. The boxes show the interquartile range, with the median value indicated by the horizontal line, and the mean value shown by a ‘+’. The whiskers indicate minimum and maximum values. Results of post hoc tests: The underlined groups did not differ significantly.

GLM showed that the density of the smallest racer goby (SF) was significantly influenced by the water current velocity and presence of a PE habitat. A similar relationship was found for fish from the MF size class. For adult fish (LF class), the water current velocity as well as the depth and presence of boulders in the substrate (habitat type BO) significantly influenced their numbers (Table 2). The selected water current velocity for the racer goby SF size class was 0.057 m s^−1^ ± 0.005. The selected current speeds were 0.039 m s^−1^ ± 0.015 S.E. and 0.022 m s^−1^ ± 0.020 S.E for the MF and LF size classes, respectively. (Fig. 4a). The selected depths for the racer goby SF, MF and LF classes were 25.5 cm ± 2.624 S.E., 24.7 cm ± 1.906 S.E. and 37.7 cm ± 4.425 S.E., respectively (Fig. 4b). The sites where the LF fish were present differed significantly in depth from sites where the fish of the other two size classes were found. No significant differences occurred in terms of water current velocity between the LF and MF sites and between the SF and MF places (Fig. 4c,d).

## Discussion

The upper Strwiąż River can be used to obtain information regarding how the racer goby, a species typically found in lower river sections, manages in the submountain river. In addition, the Strwiąż River runs the upper range edge of this species^30,35^. Species at the edge of their range are called peripheral^43^. Therefore, studying the racer goby population in this river provides additional information about the species functions at its range edge/periphery, where the persistence of a stable population is particularly difficult^44,45^. The Strwiąż River in the territory of Poland, is an example of such a peripheral basin that has survived with a good ecological status (as defined in the Water Framework Directive (WFD)^46^ because of effective land management and various forms of implemented protection (i.e., Natura 2000, landscape park)^35^. Due to the maintenance of the natural characteristics of the river channel, racer gobies in the Strwiąż River showed mosaic distribution in habitat patches meeting its requirements. The presence of fish species in a habitat patch, and thus, their distribution in the river, is most influenced by abiotic factors, such as the water current, substrate granulation and water depth^1,39,47^. Individual species have different requirements in this respect, and their mosaic distribution is thus often observed in rivers^48,49^.

The mosaic fish distribution in rivers also applies to specific size classes (usually corresponding to age) due to different requirements at various stages of an individual’s life cycle and the tendency to reduce intraspecific competition via the spatial distribution of habitats^45,49^. Evident preferences towards habitats with large submerged objects serving as hiding places were characteristic of the largest gobies. They chose places with boulders, and they were predominant in this type of habitat. A small number of smaller individuals in the habitat suggests that larger fishes won in competition with individuals of smaller sizes. Boulders with spaces in between with low water current velocity values seem to be the most hydrodynamically suitable habitat allowing for saving energy. Abiding in strong water current is costly in terms of energy, but boulders provide shelter from strong water current^50^. Behind boulders and between them, at the bottom, water current reached the zero value. Therefore, adult, largest gobies were very likely to choose the ‘boulders’ site in Strwiąż, while forcing smaller individuals to occupy places with faster water current, i.e. less suitable in terms of saving energy.

**Figure 5:**
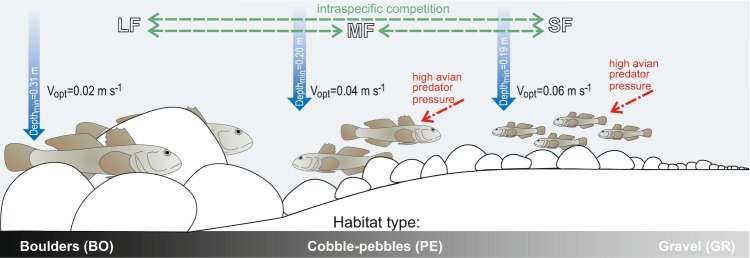
Conceptual model showing habitat preferences and relationships between racer goby size classes in the submountain river at the edge of the species range; depth_min_ – minimum depth at which racer gobies were observed; V_opt_ – optimal velocity for racer gobies.

In the Strwiąż River, larger gobies inhabit large cobbles and boulders, while in lowland rivers, racer gobies prefer bottom substrates comprising sand and fine sediments containing large, immersed objects that serve as useful hiding places^21,38^. In submountain rivers, this type of bottom substrate is not frequent. Kakareko *et al*.^21^ data on racer goby requirements suggest that a significant portion of the surveyed Strwiąż reach should be unsuitable for racer gobies, mainly due to excessively fast water current, but most likely also due to bottom substrate granulation. Depending on the body size, the species was divided between particular habitats. In the Strwiąż River, significant differences in the densities of large and small racer gobies found in areas with various bottom substrates were observed. The occurrence of large gobies was associated with boulders, while small gobies were more numerous in zones with finer bottom fractions (Fig. 5). A certain association with hard substrate was also observed *in situ* conditions by Krpo-Ćetković *et al*.^16^ in the Danube (Serbia), where the highest occurrence of the racer goby was on pebble bottom. The authors, however, did not differentiate between age classes.

Gobies respond to the presence of other individuals of their species, as well as other species^51^. Visual isolation is very important for territorial fish species. Physical structures provide visual isolation from other fish, reducing territorial needs^52,53^. These factors may be even more important than hydrodynamic-related energy savings, because it allows individuals to avoid antagonistic intra- and interspecific interactions^54^. When sufficiently suitable habitats are scarce and do not occur continuously, habitat niches occupied by different size classes can partially overlap, creating strong intraspecific competition (Fig. 5) that potentially results in ontogenetic changes in habitat selection. Large racer goby specimens are aggressive towards other fish and try to occupy the most favourable habitats for themselves^51,55^, and smaller fish were forced to occupy worse habitats. The reproductive biology of the racer goby (speleophilic species) may also be the reason why males search for appropriate places, such as niches under stones, to deposit female eggs, and the males guard the eggs until hatching^17,36^.

Predators are also a biotic factor that strongly affect the distribution of fish in rivers^56^. In the Strwiąż River, large chub (*Squalius cephalus*) and brown trout (*Salmo trutta*) are piscivorous fish^35^, and zones with shallow water offer shelter to smaller individuals against large predatory fish^57,58^. Most small-sized racer gobies were caught in the shallowest zones. On the other hand, although less exposed to attack by large predatory fish, larger gobies selected deeper places (but only where boulders and large cobbles were present, allowing hiding and forage). But although escaping to shallow water is an effective way to avoid predatory fish, the threat from piscivorous birds increases in this case. In the Strwiąż River, grey herons (*Ardea cinerea*) and black storks (*Ciconia nigra*) were often observed foraging in the shallows (Kukuła and Bylak *unpubl. data*). The black stork is a predator posing one of the serious threats to the ichthyofauna of mountain streams in the Carpathians^59^. The stork’s preference for shallow reaches of the streams with stony bottom, inhabited by bottom-dwelling fish, is linked to the visual fishing method of storks^60^.

The habitat suitabilities for racer gobies of different age classes are characterized by two environmental factors previously discussed, i.e., the type of bottom substrate and depth, verified by the water current. In experimental and field studies in lowland rivers, racer gobies avoided water currents exceeding 0.1 m s^−1^ ^38^. The water current strongly influences the availability of other environmental elements and strongly limits the space available for racer gobies. In submountain rivers, habitats especially desirable for larger individuals seem to be boulders and large cobbles (Fig. 5). At a larger geographic scale, however, considering the entire submountain river, such habitats are found mainly in zones with a water current velocity too fast for racer gobies. Therefore, the species was not found in a significant portion of the river studied^35^. Zones with more turbulent water flow occur more frequently in submountain rivers than in lowland rivers^61,62^. Kakareko *et al*.^21^ reported that in lowland rivers, racer gobies mainly reside in habitats off the main channel that have slow water velocities and both soft and hard bottoms, using various submerged objects as shelters. Few of these habitat qualities are found in submountain rivers. Therefore, racer gobies had few suitable places to inhabit in the Strwiąż River. At a larger geographic scale, the most suitable racer goby habitats occurred in patches separated from each other^35^, but the population is maintained, because the survival of the population at the edge of the species range largely depends on the heterogeneity of habitats on a small scale^35^. The presence of all size classes suggests that the species finds suitable feeding grounds, shelters, and spawning grounds, as well as places of growth of fry there.

Preservation of the river channel’s natural characteristics with the diverse habitat mosaic ensures that the population remains stable even at the edge of the species range. Therefore, protection of the species at the range edge protects not only the species itself, but also the specific genetic features of the population. Such features of peripheral populations, different than those in the centre of the range of the species, may serve as a preadaptation preceding future environmental changes^44^. The exclusion of peripheral taxa from protection programs could result in a significant loss of overall genetic resources^43^. Therefore, we believe that there is an urgent need for action to protect river fish species populations at the edge of their range. Protection of such populations is recommended, because populations functioning near the edge of the species range appear to have the highest potential for speciation due to their exposure to variable environmental conditions at the boundaries of species’ tolerances^63^. Peripheral areas of river basins, where fish species exist on the edge of their range, are often located in zones with relatively low levels of human pressure^30,35^. Due to this, the populations could have survived in good state, and are worth a more thorough insight, and consequently deserve to be included in local fish protection plans.

On the other hand, referring the obtained results to alien species, even when separated by long sections of unfavourable habitat parameters, patches of suitable habitats can form a stepping stones that allow significant extension of the species range, even to areas that are far from optimal^64^. The construction of dam reservoirs is frequently accompanied by the development of habitats suitable for gobies. Dam reservoirs are mentioned as sources of secondary invasion of the species. Dam reservoirs with shallow areas near the shores offer extensive areas with habitats suitable for the goby^65^. Until now, forecasts of e.g., racer goby expansion routes in areas wherein the fish is considered an alien invasive species have been limited to lowland rivers, particularly those on which dam reservoirs are constructed^66^. Our data showed that racer gobies also cope well in difficult environmental conditions in submountain rivers. In such rivers, relatively small patches of suitable habitats, considerably smaller than those offered by dam reservoirs, ensure fish recruitment, and in the case of invasive populations could become a source of further expansion. Therefore, our results can also be used to develop a framework for the risk assessment of alien racer gobies that may extend their range through river habitat networks. Forecasting threats related to alien gobies should include areas that seem suboptimal.

Ohayon and Stepien^19^ raised the need for further studies to assess the racer goby’s spreading abilities. Others, for the purpose of recording and monitoring of racer goby, recommended intensification of research in soft-bottom habitats as places particularly preferred by the species^67^. Recently, Kakareko *et al*.^21^ suggested that the plasticity of the species’ habitats reflects its ability to occupy sub-optimal environments, but with less preferred hard substrates. Larger gobies, however, in areas of soft substrata, were observed to excavate cavities underneath stones or pieces of wood actively creating their own refuges from elevated water velocities, and smaller ones have been seen to use small stones as shelters^21^. Our research significantly broadens the knowledge concerning the ecological abilities of this species. With regard to invasive populations, the presence of stony bottoms, quite certainly cannot be considered as a factor excluding potential colonisation by racer goby, and in submountain rivers it might be the preferred kind of bottom. The density of racer goby in the submountain river was even higher than at other sites in the zone of its native range (e.g. in the Dniprodzerzhynsk Reservoir on the Dnieper River in Ukraine^68^). It turned out that hard substrate does not preclude the functioning of a vivid, abundant, and self-sustaining population of racer goby.

## Methods

### Ethics statement

A sampling permit (No. RG-IX.7143.6.2015.MS) was issued by the Marshal Office of the Podkarpackie Voivodeship following approval by the Regional Directorate for Environmental Protection. Research project was approved by the Department of Biology and Agriculture’s Committee for Research Ethics. The research was conducted under license to operate electroshocking tools and license to perform animal investigations according to legislation on the protection of animals and the recommendations of the International Council for Laboratory Animal Science (ICLAS).

### Study area

The headwaters of the Strwiąż River are located in Poland, the Eastern Carpathians and the Sanocko-Turczańskie Mountains, and the Strwiąż River basin is part of the upper (Carpathian) Dniester River basin (Fig. 2a). The river is 94 km long, and the basin has an area of 955 km². The territory located in Poland includes the upper part of the Strwiąż River, which is 17.5 km long and has a basin area of approximately 200 km². The Strwiąż is a submountain river with a strongly variable channel: typical montane sections with rapid water currents and stony bottoms alternate with sections of slow water flow and sandy and sandy-muddy sediments. The river reach (SR, length 115 m) studied was located in the lower part of the Polish section of the Strwiąż River. The river reach covered all types of habitats occurring in the submountain portion of the Strwiąż, i.e., riffles with a stony bottom and fast flowing water, runs and pools with deeper water and a pebbly or pebbly gravel bottom. This reach of the river was selected for detailed research, because it was the highest located fragment at the upper edge of range of the species in this catchment where gobies belonging to all three size classes occurred in high abundance. Above this reach, the abundance of gobies varied from several to a dozen individuals per 100 m2 of the area of the river (Table 1), reaching zero at a distance of approximately ~6 km above the analysed river reach^35^.

### Sampling design

First, 103 line transects at right angles to the main axis of the water current spaced every 1 metre were determined using a laser rangefinder with a tripod. Each transect was stabilized in the field with wooden stakes driven into the river banks. In the designated stripes, 2 ropes were stretched between the pairs of stakes, and the stripe transect was determined. On the ropes, one-meter-long sections were marked with colourful ribbons, which allowed for the precise determination of 1194 squares (1 m x 1 m), equalling 638 sites in which racer gobies were caught (Fig. 1b). The racer gobies were caught in late summer/early autumn (from 10 to 16 September 2015), which is when young-of-the-year (YOY) fish are readily identified^69^.

Caught individuals were arbitrary divided into 3 size classes, according to size at age: small fish (SF), total length (TL) ≤ 40 mm, probably mostly YOY; medium-sized fish (MF), 40 mm < TL ≤ 60 mm; and large fish (LF), TL > 60 mm, probably mostly adults^17^.

Fish were caught using consistent methods with backpack electrofishing equipment (IG600T, Hans Grassl, GmbH, Germany; DC/AC; 650 W direct current; 1,200 W impulse current; 115–565 V). A sampling permit (No. RG-IX.7143.6.2015.MS) was issued by the Marshal Office of the Podkarpackie Voivodeship following approval by the Regional Directorate for Environmental Protection. Research project was approved by the Department of Biology and Agriculture’s Committee for Research Ethics. The research was conducted under license to operate electroshocking tools and license to perform animal investigations according to legislation on the protection of animals and the recommendations of the International Council for Laboratory Animal Science (ICLAS). Each fishing crew consisted of one person operating the anode and three people capturing and measuring the fish. To avoid startling the fish, catches were brought in every second (width of one metre) stripe transect, wading from the right to the left river bank. The catches were conducted starting at the most downstream transect. At each sample point, an anode was immersed in the centre of the square for ~10 s, a period proven effective for electrofishing^70^. The electric field parameters were adapted to the water conductivity and the physical nature of the river. The applied voltage was reduced (effective electric field of approximately 1 m diameter). It was sufficient to avoid electrical disturbance of non-sampled areas. Such point abundance sampling is particularly recommended as a useful technique for studying habitat preferences of species^71^. Caught fish were identified and then released as soon as possible after completion of processing (i.e., measuring), approximately 20 metres below the most downstream fishing transect.

Based on literature data^21^, the most important environmental factors affecting racer goby distribution are presumably the water depth, water current velocity, and type of bottom substrate. In each 1 m × 1 m square (at each site) of the sampled river reach, the substrate composition was estimated as the percentage of the area covered by different particle size fractions. Six fractions of the bottom substrate were distinguished: boulders (>256 mm), large cobbles (256–131 mm), small cobbles (130–65 mm), pebbles (64–17 mm), gravel (16–2 mm), and sand (<2 mm). This division was based on the criteria proposed by Bain *et al*.^72^

The water depth and water current velocity (~2 cm above the bottom; and in the case of boulders, between them) were measured along twenty-two line transects spaced every five metres. Measurements along the transects were made every 0.5 m, yielding 530 sampling points. The current velocities were measured using an acoustic Doppler velocimeter (Flowtracker, SonTek, San Diego, CA, USA). Spatial variations of the river water depth and velocity were estimated by the inverse distance weighting method at a resolution of 0.1 m. All spatial analyses were performed using ArcGIS 10.1 software with the spatial analysis extension^73^. In addition, water temperature, conductivity, and dissolved oxygen content were measured at 10 points along the entire river reach sampled using a multiparameter metre (6600 V2, YSI Incorporated, Yellow Springs, Ohio, USA). The characterisation of abiotic factors was done after sampling the fish in order to avoid startling the fish.

### Data analysis

Statistical data analyses were performed using STATISTICA 12 (TIBCO Software Inc., Palo Alto, CA, USA), and all multivariate analyses were performed using PRIMER v7^74^. Using cluster analysis (Ward’s linkage), sampling sites were divided into habitat types (see Supplementary Fig. S1) differing in the percentage shares of individual fractions in the substrate. Percentage data were arcsine transformed.

Using one-way permutational multivariate analysis of variance (PERMANOVA) with 999 permutations, three distinguished habitat types were compared in terms of their share of six substrate categories using the Bray–Curtis matrix of dissimilarities. Pairwise tests were used to compare the significance of differences between pairs of habitat types. The proportion of each fraction in the different habitat types was then determined using the SIMPER procedure.

One-way PERMANOVA was also used to compare three types of habitats in terms of their percentage of three distinguished racer goby size classes. Species data were log transformed [log(x + 1)], and percentage data were arcsine transformed. Because racer gobies were not found in many of the examined squares (sites), a ‘dummy’ species was incorporated for this analysis^59^. In addition, SIMPER was used to determine the contribution of each goby size class to the dissimilarity between the three habitat types.

Czekanowski’s index (CZ) was used to estimate the degree of habitat overlap between the racer goby size classes with regard to the habitat type as follows: CZ = 1 − 0.5 (∑ |A_i_–B_i_|), where A_i_ and B_i_ are the numbers of fish from the compared racer goby size classes occupying habitat type *i* divided by the total counts of both classes in this type of habitat. CZ ranges from 0 (no overlap) to 1 (full overlap), with values > 0.6 assumed to be considerable overlapping^75^.

For comparisons of water current velocities at sites where racer gobies were found and water depth, nonparametric one-way ANOVA (Kruskal-Wallis test) and post hoc tests for Kruskal-Wallis ANOVA^61^ were used. The comparison of water temperature, conductivity, and dissolved oxygen content measured in each type of habitat involved a one-way ANOVA test^76^.

The responses of racer gobies of different sizes (ages) to habitat type, current velocity, and water depth were also analysed using generalized linear models (GLM). The number of racer gobies per sample was treated as a Poisson-distributed response with a log-link function relating the racer goby abundance to the measured environmental variables^77^.

## Supplementary information

Supplementary information

## Data Availability

The datasets generated during and/or analysed during the current study are available from the corresponding author on reasonable request.
