# Supplementary material for: Habitat selection patterns of a species at the edge – case study of the native racer goby population in Central Europe
Source: Sci Rep. 2019 Dec 23;9:19670. doi: 10.1038/s41598-019-56264-7 (PMC6928068; doi:10.1038/s41598-019-56264-7)
Supplement: Supplementary file 1 — Supplementary information [file 41598_2019_56264_MOESM1_ESM.pdf]

Supplementary material

Habitat selection patterns of a species at the edge – case study of the native racer goby population in Central Europe

Krzysztof Kukuła[1], Bernadetta Ortyl[2], Aneta Bylak[1,*]

[1]Department of Ecology and Environmental Monitoring, University of Rzeszow, Zelwerowicza 4, 35-601 Rzeszow, Poland

[2]Department of Agrobiology and Environmental Protection, University of Rzeszow, Zelwerowicza 4, 35-601 Rzeszow, Poland

*Correspondence and requests for materials should be addressed to A.B. (email: abylak@ur.edu.pl) or K.K. (email: kkukula@ur.edu.pl)

Table S1. One-way PERMANOVA of Bray–Curtis distances comparing three types of habitat (BO, PE, GR) at sites in the Strwiąż River based on the arcsin of the percentage shares of six bottom substrate fractions; Monte Carlo test, 999 permutations. BO – boulders; CP – pebbles/cobbles; GR – gravel; SS – sum of squares; MS – mean square.

Source	d.f.	SS	MS	pseudo-F	P
Habitats	2	673140	336570	418.06	0.001
Residual	635	511220	805		
Total	637	1184400			
Pair-wise tests			t		P
BO vs PE			12.61		0.001
BO vs GR			25.11		0.001
PE vs GR			22.35		0.001

Table S2. Bottom substrate fractions contributing to the dissimilarity between habitat types at Strwiąż River sites (SIMPER).

Bottom substrate fractions	Habitat type			Averange dissimilarity	Contribution to dissimilarity %	Cumulative dissimilarity %
	GR Mean±s.d.	PE Mean±s.d.	BO Mean±s.d.			
gravel	76.6 ± 20.1	8.2 ± 10	3.7 ± 6.7	21.89	33.02	33.02
large cobbles	5.8 ± 8.8	39 ± 24.8	23.8 ± 14.7	12.33	18.60	51.63
boulders	4 ± 9.5	3.9 ± 7.8	52.6 ± 19.4	11.75	17.73	69.35
small cobbles	4.1 ± 6	25.5 ± 16.6	12.1 ± 11.7	9.36	14.12	83.48
pebbles	7 ± 9.5	19 ± 20.5	6.4 ± 8.5	7.88	11.89	95.37
sand	2.6 ± 6.7	4.4 ± 17.8	0.8 ± 3.4	3.07	4.63	100

Table S3. One-way PERMANOVA of Bray–Curtis distances comparing three habitats types (GR, PE, BO) in the Strwiąż River based on the percentage shares of three size categories of racer goby abundance; Monte Carlo test, 999 permutations; BO – boulder habitat, PE – pebble habitat, GR – gravel habitat (detailed habitat characteristics – Fig. A1, Table A2); SS – sum of squares, MS – mean square; Monte Carlo test, 999 permutations

Source	d.f.	SS	MS	pseudo-F	*P*
Habitat	2	11628	5814.2	9.574	0.001
Residual	635	385630	606.6		
Total	637	397260			
Pair-wise tests		t	*P*		
BO vs. PE		1.926	0.023		
BO vs. GR		1.658	0.066		
PE vs. GR		4.336	0.001		

Table S4. Shares of racer goby size classes contributing to the dissimilarity between habitat types at Strwiąż River sites (SIMPER); SF (Tl <4.0 cm), MF (Tl 4.0-6.0 cm), LF (Tl ≥ 6.0 cm), Tl – total length

Racer goby size classes	Habitat type			Averange dissimilarity	Contribution to dissimilarity %	Cumulative dissimilarity %
	GR Mean±s.d.	PE Mean±s.d.	BO Mean±s.d.			
MF	0.218±0.638	0.486±0.869	0.277±0.621	26.23	49.67	49.67
SF	0.111±0.380	0.339±0.773	0.244±0.661	16.54	31.32	80.99
LF	0.095±0.330	0.105±0.331	0.160 ±0.467	10.03	19.01	100

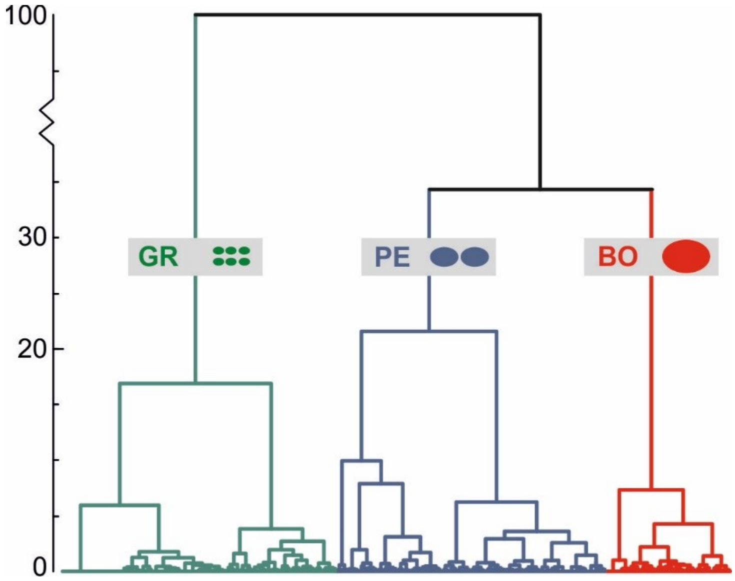

Figure S1. Dissimilarity dendrogram (Euclidean distance, Ward's method) of the sampling sites at the river reach analysed based on the arcsin of the percentage shares of six bottom substrate fractions; types of habitat: GR, gravel; PE, pebbles; BO, boulders
